# The geroprotective potential of chalcones

**DOI:** 10.1038/s41467-025-64167-7

**Published:** 2025-10-15

**Authors:** Didac Carmona-Gutierrez, Andreas Zimmermann, Guido Kroemer, Frank Madeo

**Affiliations:** 1 Institute of Molecular Biosciences, NAWI Graz, University of Graz, Graz, Austria; 2 BioHealth Graz, Graz, Austria; 3 BioTechMed Graz, Graz, Austria; 4 Team “Metabolism, Cancer & Immunity”, Centre de Recherche des Cordeliers, UMRS 1138, Inserm, UniversitéParis Cité, Sorbonne Université, Paris, France; 5 Metabolomics and Cell Biology Platforms, Gustave Roussy Institute, Villejuif, France; 6 Institut du Cancer Paris CARPEM, Hôpital Européen Georges Pompidou, France-HP, Paris, France

**Keywords:** Ageing, Autophagy, Drug development

## Abstract

Aging is the most important risk factor for multiple pathologies including cardiovascular, neoplastic, metabolic and neurodegenerative diseases. Potential geroprotective strategies involve lifestyle-related, nutritional and pharmacological interventions. Recently, chalcones, a subgroup of secondary plant metabolites, have gained attention. 4,4’-dimethoxychalcone was the first chalcone to be shown to mediate geroprotection and lifespan extension across different species. Several other chalcones also exert anti-aging effects at the cellular and organismal levels. Defined mechanistic routes that are causally involved in these protective effects have been delineated. Here, we summarize current evidence supporting the potential of 4,4’-dimethoxychalcone and other chalcones as geroprotective agents.

## Abstract (web summary)

Aging is driven by biological processes that can potentially be targeted by pharmacological interventions to delay its onset and extend healthspan. Here, the authors show the growing evidence supporting chalcones, a subgroup of secondary plant metabolites, as novel geroprotective agents

## Introduction

### Caloric restriction and caloric restriction mimetics

Aging is a complex biological process characterized by a progressive decline in physiological function, which encompasses the manifestation of a number of hallmarks^1^ and the accompanying loss of organismal health^2^. Biological aging increases the risk of developing age-associated chronic pathologies such as type 2 diabetes, cardiovascular events, neurodegenerative disorders like Alzheimer’s and Parkinson’s disease, and cancer. In recent decades, advancements in healthcare and technology have led to a remarkable rise in life expectancy. This confers individual advantages but menaces the global economy by overloading healthcare and social systems. This negative impact of population aging is explained by the fact that the increase in healthspan, the period of life free from age-related morbidities and disabilities, has not kept pace with the extension of lifespan. As a result, the incidence of age-related diseases has increased to pandemic proportions.

Crucially, the aging process involves regulatory molecular networks and is thus potentially pliable. In fact, a number of nutritional and pharmacological interventions have been proposed as anti-aging strategies, which may be able to harness this pliability, and hence postpone, decelerate or halt the aging process. Among these interventions, caloric restriction (CR), a dietary regimen that reduces calorie intake without malnutrition, has long been recognized for its ability to extend lifespan and improve healthspan across various organisms. Significantly, numerous age-related disorders that are improved by CR in model organisms have also shown effects in human studies^3^, for example, regarding obesity, type 2 diabetes, cardiovascular disease or the incidence of cancers^4^.

Mechanistically, CR activates a range of cellular pathways associated with nutritional stress, including pathways involved in stress resistance, DNA repair and metabolism. These pathways are regulated by master transcription factors (e.g., FOXO1, NRF2, PPAR-α, PGC-1α, etc.) and ultimately promote longevity^3,4^. One of the key antiaging mechanisms is autophagy (from ancient Greek, “self-eating”), a catabolic process responsible for the lysosomal degradation of subcellular components that serves as a recycling mechanism to meet nutritional demands upon nutrient deprivation. Thereby, it removes old, superficial, damaged and dysfunctional components, facilitating ‘cellular renewal’ at the molecular level. Consequently, experimental enhancement of autophagy promotes longevity, while disabled autophagy is involved in numerous diseases, including cancer, neurodegenerative disorders and metabolic syndrome^5^. The regulation of autophagy is highly conserved and governed by a number of negative and positive key regulators^6^ as well as by several autophagy-related genes (ATGs), which are crucial for the successive stages of autophagy^7^.

While CR is an established strategy to robustly promote healthspan (and possibly lifespan), achieving and maintaining CR in the long-term is challenging for humans. This is linked to the fact that classical CR strategies consist in reducing the calorie content of each meal without reducing the frequency or timing of breakfast, lunch and dinner. Periodic fasting is a more attainable strategy in which calorie intake is restricted to a specific time window^4^. Similar to CR, time-restricted, intermittent and long-term fasting can improve various health-related parameters, with autophagy serving as the key underlying mechanism^4^. Still, CR and fasting can be problematic for specific individuals, such as pregnant or breastfeeding mothers, children and adolescents, older adults, very lean persons or special disease groups.

For this reason, much effort has been invested in developing alternative strategies that induce CR-like effects without the need of reducing calorie intake. One non-invasive approach consists in the use of compounds that activate the same protective pathways as would CR. We refer to such agents as ‘caloric restriction mimetics’ (CRMs)^8^. Importantly, many if not all CRMs depend on autophagy induction to exert their beneficial effects. Some examples include rapamycin, a specific inhibitor of the negative autophagy regulator mTOR^9^, the polyamine spermidine^10^, the hypoglycemic drug metformin^11^, the glycolytic inhibitor D-glucosamine^12^, NAD^+^ precursors^13^, or acetylsalicylic acid^14^, among others^8^. Some CRMs are being evaluated in clinical trials for their capacity to mitigate are-related diseases^15^.

### Flavonoids and chalcones

One group of CRMs falls into the class of polyphenols, which - like most CRMs - are naturally occurring compounds. Polyphenols are a very diverse group of secondary plant metabolites that can be found in a variety of food items, including fruits, vegetables, tea, coffee or cocoa. Of note, their translational potential is significant because side effects in humans are rare, perhaps due to the coevolution of higher animals with polyphenol-containing plants used as a food source. Polyphenols have characteristic structural features and represent the largest group of phytochemicals. A polyphenol-rich diet reportedly decreased the risk of chronic diseases^16^, and some polyphenols, most prominently resveratrol^17^, have been characterized as CRMs^8^. The largest family within the polyphenols are the flavonoids, which share a structural backbone in which two C6 units are of phenolic nature. Several flavonoids, like epigallocatechin-3-gallate or quercetin, have been suggested to act as CRMs^8^.

**Fig. 1:**
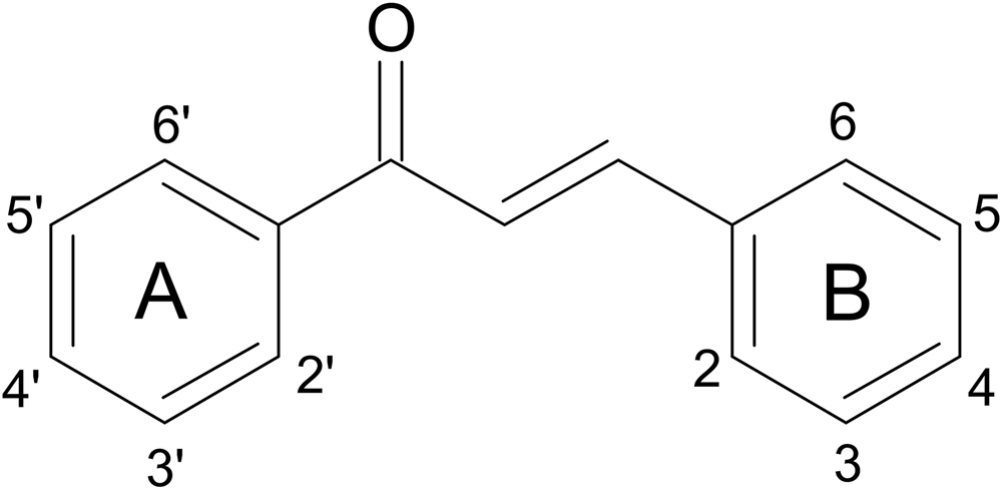
Core chemical scaffold of chalcones. Chalcones are characterized by two aromatic rings (termed as rings **A** and **B**) connected by a three-carbon alkenone moiety.

The flavonoid family is subdivided into several other subclasses, one of which are the chalcones. Chalcones are characterized by a core chemical scaffold. They are α,β-unsaturated ketones, composed of two aromatic rings (referred to as rings A and B) connected by a three-carbon alkenone moiety, also known as chalconoid^18^ (Fig. 1). The term “chalcone” originates from the Greek word “chalcos,” meaning “bronze,” owing to the typical colors exhibited by most natural chalcones. The therapeutic potential of chalcones has been acknowledged for long. A wide variety of biological activities have been ascribed to chalcones, such as antimicrobial, antidiabetic, anti-inflammatory and antiproliferative effects^19^. In fact, chalcone-rich plants have been connected to health benefits and used as therapeutic remedies across different cultures^19^. While the first natural chalcone was isolated in 1910, the first successful attempt to synthesize chalcones dates back to the 19th century. In fact, chalcones have attracted much interest with respect to their natural or artificial synthesis and are considered as privileged structures in the field of medicinal chemistry^18^. This is linked to the fact that the synthesis and chemical modification of chalcones is relatively easy.

In this minireview, we compile evidence in favor of the potential antiaging effects of chalcones. We place special emphasis on chemically defined compounds (rather than chalcone-containing plant extracts), for which (i) antiaging phenotypes have been reported in different model organisms, (ii) preferentially by several laboratories, and (iii) mechanistic evidence points towards their mode of action as CRMs.

## 4,4’-dimethoxychalcone

### Longevity effects of 4,4’-DMC

4,4’-dimethoxychalcone (4,4’-DMC) has been the first chalcone to be causally linked to geroprotective properties^20^. In a screening campaign comparing 180 different flavonoids, 4,4’-DMC emerged as the strongest hit to promote chronological lifespan (*i.e*., cell survival during aging) and to reduce the aging-associated production of reactive oxygen species (ROS) in the yeast species *Saccharomyces cerevisiae*. These markers are commonly applied to model the aging of postmitotic tissues^21^. This geroprotective capacity was validated in further aging-relevant model systems. 4,4’-DMC extended the lifespan of the nematode *Caenorhabditis elegans* as well as the fruit fly *Drosophila melanogaster*, and in different human cell lines, it promoted clonogenic survival upon prolonged culturing. In addition, intraperitoneal injection of 4,4’-DMC significantly diminished the infarction area in mice challenged with prolonged myocardial ischemia (with no reperfusion), thus establishing cardioprotective effects in an aging-relevant scenario^20^.

Notably, 4,4’-DMC was identified in the leaves and stems (but not in the roots) of *Angelica keiskei* Koidzumi^20^, a plant that has been used in Asian traditional folk medicine for millennia^22^. Incidentally, this plant is also known in Japan as “ashitaba”, meaning “tomorrow leaf”, or in Korea as “shinsuncho”, meaning “elixir of life”. *A. keiskei* Koidzumi was heavily consumed in Hachijojima (Japan), also known as the “island of longevity”. Indeed, *A. keiskei* Koidzumi extracts have been reported to have anti-carcinogenic, anti-diabetic, anti-inflammatory and anti-hypertensive properties^22^. A number of bioactive flavonoids isolated from this plant might account for these effects, including several chalcones beyond 4,4’-DMC. For instance, 4-hydroxyderricin and xanthoangelol, two of the most abundant chalcones contained in this plant, have been shown to exert effects against tumors, inflammation and diabetes^22^.

### Mode of action of 4,4’-DMC

The identification of 4,4’-DMC came along with a number of mechanistic studies. 4,4’-DMC induces autophagy in all model organisms including yeast, nematodes, flies and mice^20^. As true for CRMs^8^, the geroprotective effects of 4,4’-DMC largely depend on autophagy. The disruption of autophagy-related (ATG) genes, which are essential for autophagic flux, abrogated 4,4’-DMC-induced lifespan extension in yeast, worms and flies, as well as cardioprotection in mice^20^. Still, 4,4’-DMC may also act in an autophagy-independent manner under certain conditions^23^. For instance, 4,4’-DMC protects mice against liver damage triggered by acute ethanol intoxication, and this hepatoprotective effect does not depend on autophagy^20^. Short-term effects like this one might likely be attributed to the antioxidant properties of 4,4’-DMC, a trait shared by many flavonoids. A number of studies have delineated important structural features that contribute to oxidative radical scavenging^24^. Nevertheless, it has become evident that the idea that the health-improving effects of flavonoids solely rely on their antioxidant capacity is outdated^24–26^, and that this capacity represents just one among several mechanisms explaining their beneficial effects.

**Fig. 2:**
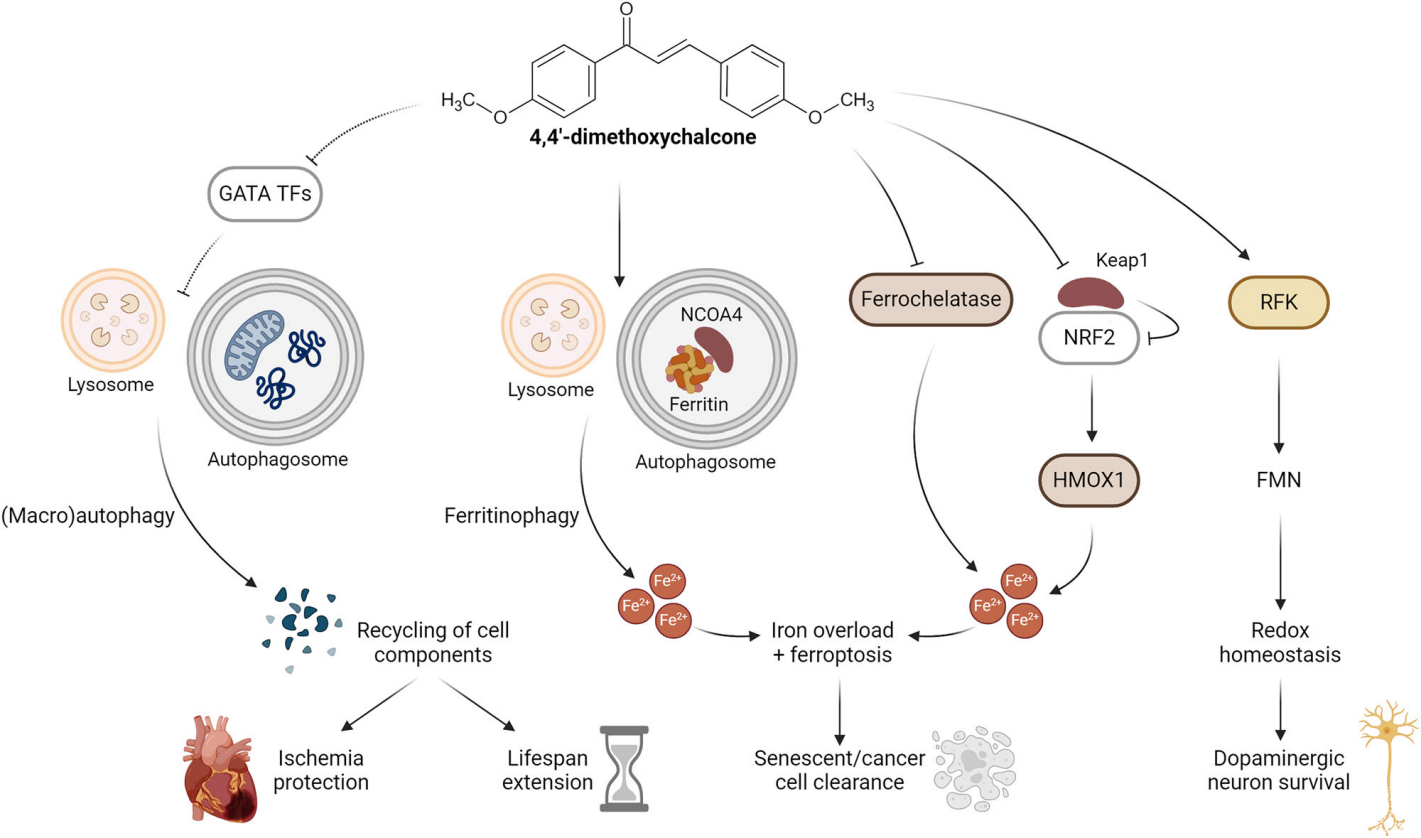
Currently known targets of 4,4’-dimethoxychalcone. 4,4’-dimethoxychalcone (i) inhibits specific, autophagy-repressing GATA TFs, (ii) interacts with iron homeostasis and (iii) promotes redox capacity both via enzymatic and scavenging activities. The engagement of these pathways results in diverse protective effects, including promotion of organismal lifespan. *TFs*, transcription factors. *HMOX1*, heme oxygenase 1. *NCOA4*, nuclear receptor coactivator 4. *NRF2*, Nuclear factor erythroid 2-related factor 2. *Keap1*, Kelch-like ECH-associated protein 1. *RFK*, riboflavin kinase. *FMN*, flavin mononucleotide. Created in BioRender. Zimmermann, A. (2025) https://BioRender.com/vj1425e.

The autophagy-inducing and geroprotective activity of 4,4’-DMC turned out to largely rely on the inhibition of GATA transcription factors (TFs) (Fig. 2). In yeast, the deletion of the yeast GATA TF Gln3 extended chronological lifespan and promoted autophagy induction^20^, suggesting that basal Gln3 activity might be a repressor of autophagy. Importantly, 4,4’-DMC seems to mimic a state of Gln3 inhibition. Accordingly, DMC-treated wildtype cells showed a metabolic profile similar to untreated Gln3-deficient mutants^20^. Moreover 4,4’-DMC could not extend the chronological lifespan of yeast cells lacking Gln3 but maintained favorable effects on the longevity of yeast cells lacking other GATA TFs such as Gat1, Dal80 and Gzf3. This dependency on specific GATA TFs could be corroborated in human cells, where 4,4’-DMC failed to induce autophagosome formation upon knockdown of GATA-2 (and to a lesser extent of GATA-3 and GATA-4), but not when other GATA TFs were depleted^20^.

### GATA transcription factors and 4,4’-DMC

In multicellular animals, GATA TFs are expressed in a tissue- and cell type-dependent manner. In hematopoietic cells, GATA-1 promotes the expression of some autophagy-associated genes while it seems to repress that of others, suggesting a complex role in autophagy regulation^27^. It has also been connected to the autophagy-promoting activity of resveratrol in an osteoblast-like cell line^28^. In other tissues, GATA-2^29^, GATA-4^30^ and GATA-6^31^ might contribute to accelerated cell senescence. Interestingly, GATA-1 and GATA-2 have been suggested to regulate differentially expressed genes that characterize several brain regions of Alzheimer’s disease (AD) patients^32^. However, the potential aging-modulatory impact of these GATA TFs needs further examination. In AD, fibrillar assemblies of amyloid-β peptides (Aβ) are thought to drive the neurodegenerative process. GATA-1 reportedly represses the expression of γ-secretase-activating protein, thus attenuating the formation of Aβ plaques^33^. In contrast, knockdown of GATA-4 in Aβ fibril-infused rats reduced amyloid plaque deposition, hippocampal inflammation and cognitive dysfunction^34^. Another study modelled cell rejuvenation based on reprogramming induced pluripotent stem cells and suggested GATA-6 as a pro-aging factor that attenuates the activity of sonic hedgehog signaling and the expression level of downstream forkhead box P1 (FOXP1)^35^. The authors reported autophagy to be increased upon GATA-6 knockdown^35^. In contrast, other GATA TFs have been connected to autophagy induction under different settings. For example, GATA-3-driven autophagy may accelerate chemically induced hepatic fibrosis in mice^36^.

In nematodes, the GATA TF elt-2, the homolog of human GATA4, might counteract aging. The levels of elt-2 decrease with aging, while elt-2 overexpression extends lifespan^37^. A lifespan-relevant target of elt-2 might be O-GlcNAc transferase, the expression of which usually diminishes upon aging, but can be boosted by elt-2^38^. The GATA TF elt-3 has also been attributed a protective function. For instance, elt-3 enhances the expression of another conserved GATA TF, egl-27, the overexpression of which extends lifespan^39^. elt-3 was also shown to mediate lifespan extension upon treatment with the flavonoid baicalein^40^. During the aging process of *C. elegans*, expression of elt-3 declines following an increase in elt-5 and elt-6, and elt-3 apparently controls a significant portion of the transcriptome changes observed in association with nematode aging^41^. Mitochondrion-targeted sulfide delivery, which exerts health span-promoting effects, can reverse this decrease in elt-3 expression levels, driving geroprotective cytoskeletal and peroxisomal transcriptomic changes^42^. Interestingly, the induction of autophagy and the extension of longevity observable in 4,4’-DMC -treated nematodes were lost upon knockdown of elt-1^20^. Other GATA TFs, however, were not assessed with respect to their potential implication in 4,4’-DMC-mediated antiaging effects.

In *Drosophila melanogaster*, the GATA TF serpent (*srp*) has been connected to autophagy induction, since *srp* knockdown suppresses lysosome-autophagosomal fusion^43^. Conversely, recent research suggests that *srp* may act as a negative regulator of dietary restriction (DR)-induced lifespan extension. Fat body-specific knockdown of *srp* extended fruit fly survival, although less than DR, and these effects were further improved upon whole-body knockdown, though at the expense of a reduced fecundity^44^. In contrast to *srp*, GATAe, a TF involved in the maintenance of intestinal stem cells, seems to be required for DR-mediated longevity. Intestinal knockdown of GATAe reduced lifespan and also diminished the lifespan-extending benefits of DR^44^.

Altogether, the different GATA TFs affect the aging process in a variegated fashion. Although they participate to the aging-modulatory effects of CR/DR and 4,4’-DMC, this participation is not uniform.

### Other protective effects of 4,4’-DMC

4,4’-DMC has been shown to improve the fertilization ability and developmental potential of oocytes undergoing postovulatory aging (POA) from mice, at least in vitro^45^. This may be important in the context of in vitro fertilization, where POA limits the success rate. In particular, 4,4’-DMC counteracts a number of hallmarks of POA, including excessive ROS levels, abnormal distribution of mitochondria and increased cellular loss due to apoptosis. Accordingly, 4,4’-DMC improved fertilization and blastocyst formation rates^46^. 4,4’-DMC-induced autophagy seems to play a role in this scenario, at least with respect to ROS production. However, the role of autophagy in enhancing fertilization rates and early embryonic development has not yet been clarified. In a mouse model of traumatic brain injury (TBI), DMC was shown to improve neurological impairment, specifically learning and memory as well as motor function deficits. Interestingly, DMC administration seems to reduce neuronal apoptosis and suppress microglial activation, altogether mitigating TBI-induced neuronal tissue damage and the associated inflammatory response. These effects might be exerted through the TREM2/PI3K/AKT/NF-κB pathway^47^. It will be interesting to follow whether these mechanistic signals may be partly involved in other (neuroprotective) activities of DMC.

Recently, 4,4’-DMC has been described as a senolytic^48^. Senolytics are drugs that selectively eliminate senescent cells, which are characterized by an irreversible cell cycle arrest, resistance to apoptosis and the continuous secretion of pro-inflammatory factors. Senescent cells accumulate during aging in different tissues and drive chronic inflammation, which underlies many aging-associated diseases^49^. Reportedly, 4,4’-DMC selectively eliminates senescent cells both in vitro and in vivo. In 20-month-old mice, repeated intraperitoneal 4,4’-DMC injections for 2 months reduced the number of senescent hepatocytes, decreased the mRNA levels of multiple pro-inflammatory cellular senescence-associated factors, prevented hair loss and improved motor coordination. 4,4’-DMC alone exerted these effects, which were further improved when 4,4’-DMC was combined with the tyrosine kinase inhibitor dasatinib or the flavonoid quercetin^48^. The combination of quercetin and dasatinib was previously identified to have senolytic potential^50^. Mechanistically, in this experimental setting, 4,4’-DMC seems to induce ferritinophagy, a process in which nuclear receptor coactivator 4 (NCOA4) mediates autophagic degradation of ferritin and induces ferroptosis^48^, an iron-dependent form of regulated cell death characterized by excessive lipid peroxidation (Fig. 2). Ferritinophagy and ferroptosis are generally impaired in senescent cells^51^. Intriguingly, ferritinophagy and ferroptosis have been implicated in major diseases including cancer and neurodegeneration^52^. For instance, some cancer therapies can induce ferroptosis to suppress tumor growth^53^, and α-synuclein, a neuronal protein connected to Parkinson’s disease (PD), impairs ferritinophagy^54^. It will be interesting to study 4,4’-DMC -induced ferritinophagy in the context of these diseases.

Of note, 4,4’-DMC was found to inhibit the proliferation of different cancer cell lines via induction of ferroptosis^55,56^. Reportedly, 4,4’-DMC synergistically promotes the accumulation of labile ferrous iron (i) through upregulation of heme oxygenase 1 (HMOX1), which liberates free iron from heme and (ii) via direct binding to and inhibition of ferrochelatase, altogether resulting in iron overload^55^ (Fig. 2). Interestingly, the *HMOX1* gene is transactivated by the transcription factor NRF2, which is negatively controlled by Keap1-dependent degradation. 4,4’-DMC treatment decreased Keap1 levels, which was dependent on the ubiquitin proteasome system (UPS) but not on autophagy. Autophagy-independent 4,4’-DMC effects have been suggested previously, depending on the cell-physiological context^20^. It will be interesting to see whether this putative capacity to differentially induce the two main degradation systems (autophagy and UPS) can be harnessed for therapeutic purposes.

NRF2, a master TF enhancing the expression of antioxidant genes, and its main negative regulator Keap1 play a pivotal role in maintaining intracellular redox homeostasis^57^. Ultimately, NRF2 also contributes to anti-inflammatory responses, for instance, through direct transactivation of HMOX1. HMOX1 catalyzes the rate-limiting step of oxidative heme degradation, resulting in several bioactive products that are important for inflammatory control^58^. Importantly, the NRF2-HMOX1 axis has been specifically connected to the regulation of the NF-κB pathway^59^ and macrophage metabolism^60^. Given the pivotal importance of inflammation and immunosenescence in aging and aging-associated diseases^61^, inducers of NRF2-HMOX1 hold extensive therapeutic potential. However, some hurdles remain to be overcome, including possible off-target effects, bioavailability and safety issues^57,58^. Irrespectively, the capacity of 4,4’-DMC to upregulate HMOX1 via NRF2 activation^55^ warrants further investigation. In fact, a number of natural and synthetic chalcones have been connected to NRF2 activation in the context of various diseases^62^. This also includes natural dimethoxy-derived variants like 2’,4’-dihydroxy-3,4-dimethoxychalcone or 2’,3’-dihydroxy-4’,6’-dimethoxychalcone. Intriguingly, upon intracerebral administration, the latter chalcone suppressed the death of dopaminergic neurons in a chemically-induced PD mouse model^63^. 4,4’-DMC has shown similar effects in thus far that 4,4’-DMC mitigated motor deficits, α-synuclein aggregation and neuronal death in the *substantia nigra* in a mouse model of PD^64,65^. Here, 4,4’-DMC, which does not cross the blood brain barrier^65^, was subjected to a galenic reformulation to incorporate it into nanoparticles conjugated to a brain barrier-penetrating peptide. This strategy of 4,4’-DMC delivery into the brain restored redox homeostasis by promoting riboflavin metabolism via increased expression of riboflavin kinase, which generates neuroprotective flavin mononucleotide^66^ (Fig. 2).

Altogether, these results underline the possibility to use 4,4’-DMC for the prevention or treatment of a large panel of age-related diseases.

## 3,4-dimethoxychalcone

**Fig. 3:**
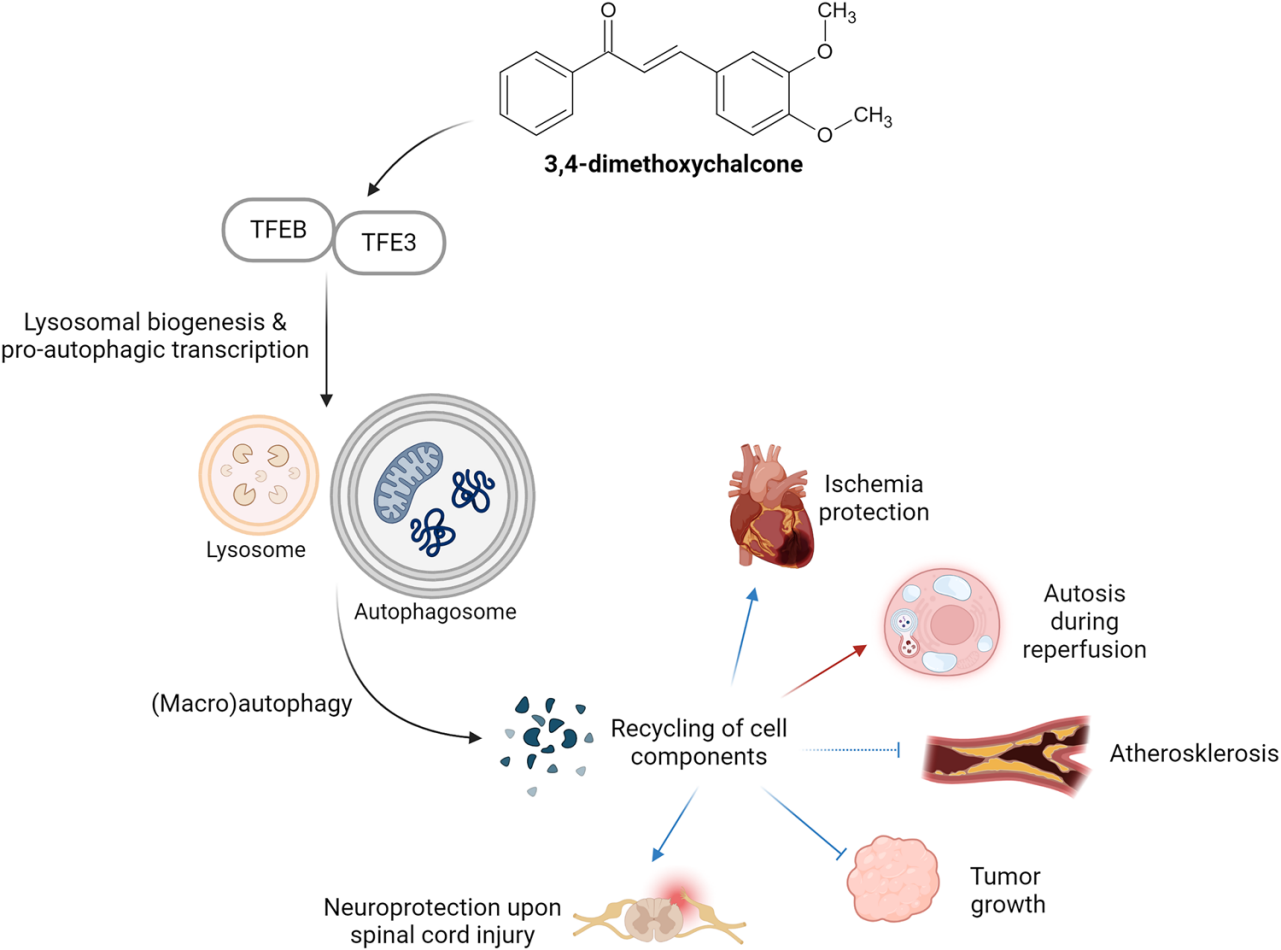
Currently known targets of 3,4-dimethoxychalcone. 3,4-dimethoxychalcone promotes lysosomal biogenesis and autophagy via two specific transcription factors, eventually promoting protective effects in different tissues. *TFEB*, transcription factor EB. *TFE3*, transcription factor binding to IGHM Enhancer 3 (TFE3). Created in BioRender. Zimmermann, A. (2025) https://BioRender.com/auoinz4.

A stereoisomer of 4,4’-DMC, 3,4-dimethoxychalcone (3,4-DMC), also stimulates autophagy and promotes good health. 3,4-DMC emerged as the best hit in a screen involving human cells that was designed to identify stimulators of autophagic flux and cytoplasmic protein deacetylation^67^, which are commonly induced by CRMs^8,68^. The pro-autophagy effects of 3,4-DMC depended on the transcription factor EB (TFEB) and the transcription factor binding to IGHM Enhancer 3 (TFE3)^67^, which are well known to favor lysosomal biogenesis and autophagy^69,70^ (Fig. 3). Double knockout of both transcription factors efficiently reduced 3,4-DMC-elicited autophagic flux and expression of key autophagy genes^67^. Intriguingly, double knockout of TFEB and TFE3 did not influence 4,4’-DMC-mediated autophagy induction; in turn, knockdown of the specific GATA transcription factors that preclude 4,4’-DMC-mediated autophagy failed to interfere with autophagy induction by 3,4-DMC.

Mice receiving intraperitoneal 3,4-DMC injection exhibited increased autophagic flux in the heart and liver. In the context of cardiac ischemia/reperfusion, 3,4-DMC treatment reduced the relative volume of the myocardial infarction in an autophagy-dependent manner^67^. It should be noted that the role of autophagy on ischemia and reperfusion might be time-dependent: protective during ischemia and the early phase of reperfusion but detrimental during the late phase of reperfusion^71^. Accordingly, the cardioprotective effects of TFEB induction via 3,4-DMC treatment might not apply to the late phase of reperfusion. Indeed, in that phase, TFEB activation promotes cardiomyocyte autosis - an autophagy-dependent non-apoptotic form of cell death – and 3,4-DMC treatment aggravates myocardial injury^72^. Of note, a recent study addressed the protective effects of 3,4-DMC in limb ischemia/reperfusion injury and showed that protection is partly elicited through TFEB-mediated activation of autophagy^73^.

In mouse models of fibrosarcoma and non-small-cell lung cancer, 3,4-DMC had no effect as a standalone treatment but improved the reduction of tumor growth elicited by the chemotherapeutic drugs mitoxantrone or oxaliplatin. This chemotherapy-improving effect was lost when cancer cells were manipulated to remove TFEB/TFE3 or when T lymphocytes were removed from the system^67^. These observations are reminiscent of some established CRMs, which have been shown to improve the capacity of chemotherapeutics to induce therapeutically relevant anticancer immune responses, provided that cancer cells are autophagy-competent^74^.

Finally, 3,4-DMC has been described to mediate beneficial effects on several mouse models of human disease. 3,4-DMC was able to reduce glial scar formation and motor neuron death while promoting functional recovery after spinal cord injury in mice. These effects were lost when TFEB was knocked down in the spinal cord^75^. Furthermore, 3,4-DMC showed antiatherogenic activity in two different mouse models of atherosclerosis, which was accompanied by autophagy induction^76^. 3,4-DMC also protected the skin against ultraviolet-A irradiation^77^. It remains to be determined, to which extent these effects can be explained by antioxidant activity or the induction of autophagy, knowing that autophagy enhancement does reduce ultraviolet radiation-mediated photoaging^78^.

Altogether, these findings support the idea that 3,4-DMC exerts broad antiaging effects that may be explained through the induction of autophagy. In this context, it appears intriguing that 3,4-DMC and 4,4’-DMC promote autophagy via separate routes of transcriptional control, namely, activation of TFEB/TFE3 for 3,4-DMC and inhibition of GATA transcription factors for 4,4’-DMC. Thus, it is plausible, yet remains to be demonstrated, that these two chalcones might be advantageously combined to optimally enhance autophagy.

## Other chalcones

A large body of evidence obtained in vitro and in vivo has connected numerous chalcones to the mitigation of age-related diseases. Several chalcones protect mice against cancer, as extensively reviewed elsewhere^79^. Furthermore, several chalcones have been shown to exhibit aging-relevant anti-inflammatory effects. Butein, for example, promotes neuronal cell viability in a lipopolysaccharide (LPS)-induced cell culture model of neuroinflammation^80^, and isoliquiritigenin reduces brain damage and attenuates motor and cognitive impairments in a rat model of TBI^81^. Moreover, isobavachalcone induces autophagic flux in a TFEB/TFE3-dependent manner and improves the outcome of immunogenic chemotherapy against established tumors in mice^82^. In a rat model of diabetes, isobavachalcone ameliorates renal damage^83^, and in a mouse model of PD, it mitigates neuroinflammation^84^. Isobavachalcone reduced Aβ aggregation in a cellular model of AD^85^ and protected cultured skeleton muscle cells against tumor necrosis factor-α-induced atrophy^86^.

Hydroxysafflor yellow A (HSYA), a chalcone glycoside present in safflower (*Carthamus tinctorius*), reportedly has geroprotective effects^87^. Like several other chalcones, HSYA protects against photoaging in thus far that skin damage induced by UV radiation in depilated mice was reduced upon topical application of HSYA^88^. Although these effects have been attributed to its anti-oxidative properties, HSYA has been shown to activate autophagy in cultured human cells and mice^89^. Furthermore, in a rotenone-induced mouse model of PD, HSYA promoted α-synuclein clearance^89^ and improved motor function^90^. Similar neuroprotective effects were observed in PD induced by the Parkinsonian toxin MPTP^91^. HSYA reduces LPS-induced neurotoxicity and neuroinflammation in dopaminergic neurons^92^. Further neuroprotective effects have been observed in models of cerebral ischemia reperfusion-injury^66,93^ and vascular dementia^94^. Moreover, in mouse models of myocardial ischemia/reperfusion injury and D-galactose-induced aging, HSYA administration alleviates heart^95^ and liver damage^96^, respectively. Notably, other chalcones like 4,4’-DMC, 3,4-DMC (see above), butein or xanthohumol also exert cardio- and/or hepatoprotective effects in vivo^97,98^.

Another group of chalcones with documented anti-aging potential are the licochalcones, found in licorice root (*radix glycyrrhizae*). For instance, licochalcone A reduces microglial activation and dopaminergic neurodegeneration while improving behavioral impairments in an LPS-induced rat model of PD^99^. In middle-aged mice, licochalcone A injection improves their cognitive ability and cerebral blood flow, possibly through immune-modulating activities^100^. Licochalcone A also exerts anti-obesity effects^101^. Thus, intraperitoneal injections of high-fat diet-induced obese mice with licochalcone A improved a number of obesity-associated markers compared with control animals, including decreased body weight, hepatocyte steatosis and fasting glycemia, among others^102^. Such anti-obesity effects may extend to other licochalcones, a series of other natural chalcones, and several synthetic chalcones. For instance, a dimethyl- and trimethoxy-substituted chalcone derivative reduces food intake, glucose intolerance, and hepatic steatosis in a mouse model of obesity^103^. Two halogen-containing 4‘-methoxychalcone derivatives, which are structurally closely related to 4,4’-DMC and 3,4-DMC, prevent body weight gain as well as deficits in glucose tolerance and insulin resistance in high-fat diet-induced obese mice. In vitro experiments revealed that these chalcones activate AMP-activated protein kinase (AMPK)^104^, a major positive regulator of autophagy. Similarly, the anti-obesity effects of licochalone A have also been mechanistically linked to AMPK activation^102^. Thus, it would be interesting to see whether these effects are connected to autophagy induction.

Besides the studies on 4,4’-DMC (see above), reports addressing possible activities of specific chalcones on organismal lifespan remain rare. One study performed in *C. elegans* showed that the dihydrochalcone aspalathin, a major active ingredient of rooibos (*Aspalathus linearis*), has antioxidative properties and also promotes the expression of the superoxide dismutase *sod-3* and that of the sole ortholog of the FOXO family of transcription factors *daf-16*. Notably, continuous feeding of aspalathin extended lifespan of *C. elegans* in a dose-dependent manner, albeit only under high glucose conditions^105^.

A screen designed to identify drugs that confer oxidative stress resistance to human primary-fibroblasts identified a chloro- and benzimidazole-substituted chalcone as one of the top hits among over 100,000 small molecules^106^. This chalcone dubbed Gr-4D exhibits no obvious cell toxicity and extends *C. elegans* lifespan by up to 50%. In human cell cultures, Gr-4D induces the expression of different NRF2-regulated genes as well as that of sestrin 1 (SESN1). Since both NRF2 and SESN1 are involved in antioxidative responses^107^, this outcome is consistent with the objective of the original screen. However, the cytoprotective activity of the chalcone was largely retained upon NRF2 siRNA knockdown, suggesting that it does not rely on NRF2 activation^106^. Whether SESN1 is involved in the prolongevity effects of Gr-4D has not been determined. However, studies in various model organisms (including mice) indicate that sestrins contribute to the regulation of aging pathways^108^. In human cells, Sestrin-2 induces autophagy^109^. In *C. elegans*, RNAi-mediated inhibition of SESN1 expression shortens, while SESN1 overexpression promotes lifespan^110^.

In sum, beyond their effects against age-related diseases, various chalcones exemplified by aspalathin, 4,4’-DMC and Gr-4D increase the lifespan of *C. elegans*. It will be interesting to see whether such pro-longevity effects extend to higher animals as well.

## Limitations, clinical potential and sex-specific responses

Taking together the above evidence, the geroprotective potential of chalcones is based on the observed efficacy across various model organisms, all of which share a crucial trait for their legitimate application: the strong evolutionary conservation of aging-associated cellular and molecular pathways. Still, each model has its distinct limitations and advantages. The genetic tractability of *S. cerevisiae* allows for comparably simple dissection of mechanistic features upon compound treatment. This includes the possibility of rather rapid screening of aging-related genes and pathways due to its short lifespan. However, it lacks the tissue complexity, endocrine systems, and immune functions of multicellular animals, precluding assessment of some translational aspects^111^. Similarly, *C. elegans*, while representing a more complex multicellular organism with differentiated tissues, also has a short lifespan and well-mapped genome. Nonetheless, its simple physiology and lack of complex organs like a circulatory system or adaptive immune system can restrict the relevance of certain findings^112^. The use of *D. melanogaster* combines a relatively short lifespan with more complex tissues, including a brain and a heart-like structure. It can be used to study behavioral aspects of aging and has advanced tools for tissue-specific gene manipulation. However, some fundamental differences in physiology, like its open circulatory system^113^ may pose challenges in modeling human responses to CRMs. One general disadvantage of all the above models is the assessment of pharmacodynamics: drug absorption and metabolism can differ markedly from mammals. In that sense, the use of mice provides the closest approximation of human aging in common preclinical research to help bridge the gap to clinical potential. Mice possess similar organ systems, metabolic processes, and immune functions, making them valuable for evaluating the systemic effects of CRMs. However, besides the technical, economic and ethical hurdles, several aspects like genetic homogeneity of laboratory animals and controlled (external trigger-free) experimental conditions represent key limitations^114^, since they contrast sharply with the diverse, multifactorial health conditions seen in human populations. Still, mice remain the gold standard for pharmacological testing prior to clinical endeavours. In that sense, mouse studies on the geroprotective effects of chalcones, especially regarding longevity, remain limited and further work will be needed to address systemic effects in mammals.

The clinical potential of chalcones as geroprotective agents will not only be subject to their long-term efficacy, but also to their pharmacokinetic characteristics, including favorable absorption, distribution, metabolism and excretion as well as to their safety profile. Natural chalcones are part of many traditional human diets and seem to be considered generally adventageous regarding toxicity and side effects^115^, although each compound certainly needs to be assessed individually. Still, a number of CRMs exemplify that in the course of translational efforts, limitations may arise that need to be tackled. For instance, the bioavailability of resveratrol is low, with rapid metabolism and excretion limiting its systemic concentrations^116^. This galenic problem seems to be common to other polyphenols, underscoring the importance of searching for strategies and formulations that at least partly reduce this limitation. For example, micronization technology increases the surface area of a particle and allows to decrease its size, potentially promoting drug bioavailability^117^. Specific adjuvants may also be advantageous: for example, the poor bioavailability of another polyphenol, the potential CRM curcumin, can be significantly increased by piperine^118^, a compound found in black pepper. Another possibility can also involve adapting administration strategies. For example, rapamycin carries immunosuppressive properties^119^ and has been linked to insulin resistance and hyperlipidemia^120^. In this case, intermittent administration has been proposed to circumvent these limitations^121^. Metformin is generally considered to have a favorable safety profile, but it can cause gastrointestinal discomfort in some individuals. Here, several approaches have been proposed to mitigate these side effects, including the use of extended-release and delayed-release strategies^122^. Altogether, these examples show that – beyond demonstrating efficacy in humans – translational steps for the use of chalcones as geroprotective agents may be challenged by pharmacokinetic and safety constraints. Thus, exploring strategies to optimize dosing regimens or develop analogs with improved therapeutic windows will likely accompany further clinical development of chalcones as potential CRMs. Nevertheless, a limited number of clinical trials do exist on specific chalcones. For example, hesperidin methylchalcone, at least in combination with Ruscus extract and vitamin C, seems to be well tolerable and effective in the frame of chronic venous disease^123^. Similar results were obtained for preparations containing licochalcone A regarding different inflammatory skin conditions^124^. Furthermore, xanthohumol was positively assessed for safety and tolerability, and its anti-inflammatory properties are currently being evaluated in patients with Crohn’s disease^125^.

One intriguing aspect upon clinical evaluation are sex-specific physiological responses. With respect to chalcones connected to geroprotection, the preclinical data in rodent models so far shows that effects are present in both male and female animals. This is the case, for example, regarding anticancer activities of xanthohumol^126,127^ or cardamonin^128,129^, among others. For other geroprotective effects, most studies in rodent models have concentrated on male animals. This includes the PD-protective potential of HSYA^89–91^ and isobavachalcone^84^ as well as other effects of HSYA, including cardio- and hepatoprotective activities^95,96^ and against vascular dementia^94^. Further examples include the activities of 4,4’-DMC^20^ and 3,4-DMC^67^ against prolonged myocardial ischaemia and 4,4’-DMC against hepatotoxicity^20^ as well as of xanthohumol against age-associated liver alterations^97^ and the anti-obesity potential of licochalcone A^101,102^. In turn, female animals were used to assess protective effects of HSYA against skin photoaging^88^ as well as neuroprotective activities of licochalcone A against PD^99^ and age-associated cognitive decline^100^. In summary, the broad range of protective effects exerted by different chalcones may be applicable to both sexes. However, more studies with female animals are necessary and comparative analyses, which are currently lacking, would be needed to determine whether some of these effects may be more pronounced in one sex or the other.

In this context, it is important to note that chalcones, including several ones that have been mentioned in this review, act as estrogen receptor (ER) modulators^130^. For example, butein^131^ and isobavachalcone^132^ exert estrogenic activities via both ERα and Erβ, while licochalcone A is anti-estrogenic^133^. Others, like isoliquiritigenin, exhibit biphasic estrogenic actions^134^, *i.e*., they act estrogenic at low and anti-estrogenic at high concentrations. Interestingly, several chalcone derivatives have also been connected to anti-androgenic activities^135,136^. Beyond receptor-modulatory effects, several chalcones also seem to interact with androgen and estrogen biosynthetic pathways. Two important enzymes involved in these pathways are the 17β-hydroxysteroid dehydrogenase AKR1C3, which catalyzes the conversion of androstenedione to testosterone and of estrone to estradiol, and the cytochrome P450 family member CYP19A1 (aromatase), which catalyzes the conversion of androgens (androstenedione and testosterone) into estrogens (estrone and estradiol). Several chalcones have been shown to inhibit AKR1C3^137,138^ or aromatase^137^, including isoquiritigenin^139^ and butein^140^. Thus, the efficacy and safety profile of chalcones might exhibit sex-specific variations. Of note, the dualistic view of estrogens as “female” hormones and “androgens” as male hormones is inaccurate, and both classes of steroids regulate critical pathophysiological circuitries in both males and females^141^. Therefore, chalcone-mediated modulation of sex steroid activity might have an impact on both sexes, independently of whether the effect involves androgenic or estrogenic pathways.

## Conclusions

Pharmacological strategies aimed at combating aging and age-related diseases are gaining significant attention as promising geroprotective approaches. In this regard, recent evidence has highlighted the remarkable potential of the flavonoid subfamily of chalcones. Several studies have demonstrated anti-aging effects of specific chalcones in various preclinical models, revealing effects on lifespan and healthspan in various models of age-related diseases. Nevertheless, data from mammalian models remain limited, especially regarding longevity and sex-specific responses. As seen with other CRMs, issues such as bioavailability, safety, and long-term efficacy will also need to be more closely addressed to substantiate the potential for future clinical application. Mechanistic studies suggest commonalities in the mode of action of a diverse array of chalcones, among which antioxidant and pro-autophagic activities stand out. However, the proximal targets/receptors of chalcones have not been identified and the pathways activated by chalcones (*e.g*., the transcription factors from the GATA and TFEB families, the redox homeostasis-regulatory transcription factor NRF2, induction of sestrins, ferritinophagy) may be specific to particular chalcones. These findings do not only encourage the search for additional natural chalcones but also support the development of more potent semisynthetic variants. Chalcones are particularly advantageous, since they are comparably easy to produce and modify, thus supporting future efforts in designing structure-activity relationships, especially with respect to hit-to-lead optimization of affinity and selectivity. This may also help to improve pharmacokinetic parameters (absorption, distribution, metabolism, and excretion). Future will tell which chalcone(s) and combinations thereof will enter pharmacological development.
